# Surgical Versus Sequential Hybrid Treatment of Carotid Body Tumors

**DOI:** 10.1515/med-2019-0115

**Published:** 2019-12-26

**Authors:** Bruno Amato, Rita Compagna, Anna Florio, Francesca Calemma, Aldo Rocca, Francesco Salzano, Sergio Brongo, Vincenzo Gasbarro, Giovanni Aprea

**Affiliations:** 1 Department of Clinical Medicine and Surgery – University Federico II of Naples, Italy – via S. Pansini, 5 - 80131 Naples, Italy; 2 Interuniversity Center of Phlebolymphology (CIFL), International Research and Educational Program in Clinical and Experimental Biotechnology, Catanzaro, Italy; 3 Department of Cardio-thoracic and Respiratory Sciences - University of Campania "Luigi Vanvitelli", Naples - via S. Pansini, 5 - 80131 Naples, Italy; 4 Unit of Vascular Surgery, S. Anna Hospital, Via Aldo Moro 8, Ferrara, Italy; 5 Department of Medicine, Surgery and Dentistry, University of Salerno, Salerno, Italy

**Keywords:** PBF, proliferation, apoptosis, AKT/mTOR, Wnt3a/β-catenin

## Abstract

Carotid body tumor (CBT) are slow-growing tumors that develop in the cervical region at the carotid bifurcation. . In a randomized study, 33 patients were treated for CBT excision: 10 patients performed preoperative embolization (PE) and 23 were treated only by isolated traditional surgery (N-PE). The first group includes patients undergoing preoperative embolization. The second group of patients (N-PE) included 11 males and 12 females. Intraoperative complications were lower in patients treated with a hybrid procedure (PE): sections of the cranial nerves were recorded in 7% of cases compared to 12% of the surgical procedure (P-value = 0.72); while the reversible nerve lesions (P value = 0.21) and the permanent ones (P value = 0.46), were instead similar in both procedures. The comparative blood loss during the operative procedure shows a P-value of 0.02. Operating times, reversible damage of the cranial nerves , incidence of stroke (0% vs1%, P value> 0.99) and post-operative hospital stay (4.1 vs. 4.2 days, P value = 0.91) did not show differences in the two groups of patients. The analysis of the results detects pre-operative embolization of CBT in reducing intraoperative blood loss and resection of the cranial nerves..

## 1 Introduction

The carotid body tumor, also known as chemodectoma or paraganglioma is a tumor that originates from the chromaffin cells of the carotid body, an organ located at the carotid bifurcation, responsible for regulating blood pressure through a system of baroceptors [1,2].

About its incidence, the glomus tumor predicts a men-to-woman ratio of 4:1, with age of onset between 50 and 75 years [3]. The incidence of multiple paragangliomas is about 9% in sporadic forms, but rises to 30-35% in familial forms. In 5% of cases the neoplasms are bilateral; this percentage exceeds 70% in family forms [4,5]. The transmission, autosomal dominant, occurs through the paternal way with the involvement of two genes: SDHD subunit D and SDAC subunit C [6]. The familiar forms can also produce catecholamines. Functional paragangliomas can affect the APUD system or associate with intra-adrenal pheochromocytoma and fall within the group of familial syndromes such as neurofibrosis [7], von Hippel-Lindau syndrome [8], Carney’s triad [9,10] and the MEN2 [11, 12]. Carotid body tumors represent the 60-78% of all paragangliomas in the head and neck district, and are characterized by slow growth and a low rate of malignancy (approximately 2-8%) [13]. The risk of adrenergic crisis, for the release of catecholamines, the possibility of compression or infiltration of the carotid vessels, and consequent risk of stroke and distant metastases (5% of the cases described in the literature), make, however, surgical removal generally appropriate, in absence of contraindications.

The therapy reserved for these tumors therefore involves surgical resection, both for the risk of local complications related to the size of the tumor in its evolution, and for the limited, but real, risk of malignancy of this pathology. Surgical resection is burdened by possible complications of nerve injuries in the neck (18-37% of cases in the literature): this can happen especially when the swellings reach critical size. Post-operative deficits of cranial nerves are generally transitory, but they are sometimes permanent with stable deficiencies in 2% to 40% of the cases involved, according to the various authors [14, 15, 16].

Preoperative embolization, through interventional radiology techniques, has been recently introduced to facilitate the resection of large tumors and decrease the risk of blood loss and cranial nerve injuries. Data on the efficacy of this technique have so far been conflicting, but they generally describe an advantage of the preoperative embolization procedure, even if the literature is actually limited both for the small number of cases of this pathology and for the number of centers that have applied this method so far, also finally considering the costs of the additional procedure.

The chemotherapeutic approach, as well as radiotherapy, are described as alternative therapeutic supports in the treatment of carotid body tumors if its dimensions are small and adjacent structures or organs are not invaded.

When CT highlights the need to resort to a surgical treatment, there are two important classification schemes, which the surgeon often refers to.

To minimize the injury of the cranial nerves, Hallet [17] proposed that the surgical dissection should fundamentally considers three anatomical zones:

– zone I, includes the common carotid, the bifurcation and the neighboring vagus nerve;
– zone II, includes the external carotid, the overlying hypoglossal nerve, the underlying superior laryngeal nerve and more superficially the mandibular part of the facial branch;
– zone III, is delimited by the internal carotid and the confluence of the cranial nerves in the deep cervical lateral plexus. Serious neurovascular lesions may occur in this area.

The classification proposed by Shamblin [18] takes instead into greater consideration the anatomical relations that the carotid body tumor contracts with the adjacent organs, classifying them into three types:

– type I, in which the tumor dislocates the two carotid branches and does not take contact with the nerves.
– type II, in which the tumor adheres to the adventitia of the vessels and nerves: they are in close contact with the neoplastic capsule and can be compressed
– type III, in which the tumor incorporates vascular-nervous structures, it is of considerable size (> 5-6 cm) and can extend up to the cranial base.

## 2 Surgical resection techniques

Surgical resection of a carotid body tumor is performed in 69% of cases by vascular surgeons and in 31% of cases by neurosurgeons, flanked by an otolaryngologist and a maxillofacial surgeon; this is necessary because of the proximity of the specificity of the structures involved [13].

The patient is subjected to general anesthesia, possibly with monitoring of brain activity through an intra-operative electroencephalogram (EEG) or brain-evoked potentials [8].

In cases of bilateral body tumor, the otolaryngologist will monitor the recurrent laryngeal nerve, especially if the vocal versus lateral string is paralyzed. Larger carotid body tumors localized above C2, require tracheal nose intubation and, if necessary, subluxation of the mandible [12].

The parotid gland is mobilized; according to the occurrence, the facial nerve is identified and isolated, and the bifurcation of the digastric muscle is identified.

The most commonly used incision is the one on the anterior edge of the sternocleidomastoid muscle. During the dissection the vagus and hypoglossal nerve are respectively identified and then isolated [5].

The subsequent separation between the carotid sheaths and the pharynx, medially localized, allows isolation of the superior laryngeal nerve that passes deeply at the level of the carotid bifurcation. Then the common and internal carotid artery are isolated and subsequently the resection of the body tumor is generally performed in the cranio-caudal direction.

Bipolar diathermic coagulation is used to isolate the carotid bifurcation and near the nerves.

For tumors extended in the parapharyngeal space, the isolation of the main branches of the facial nerve, of the glossopharyngeal and of the sympathetic cervical branch is obviously required.

The cranial nerves involved in the tumor are entirely removed and any other ipsilateral paraganglioma is removed at the same time.

If clamping of the carotid artery is required, intravenous heparin is administered in advance.

The use of a carotid artery shunt is predicted if EEG changes are recorded during clamping of the common or internal carotid. In some cases, the intervention involves a vascular graft with a saphenous vein, or a vascular prosthesis in the event that a section of carotid artery is also removed.

## 3 Hybrid treatment in vascular surgery

**Figure 1:**
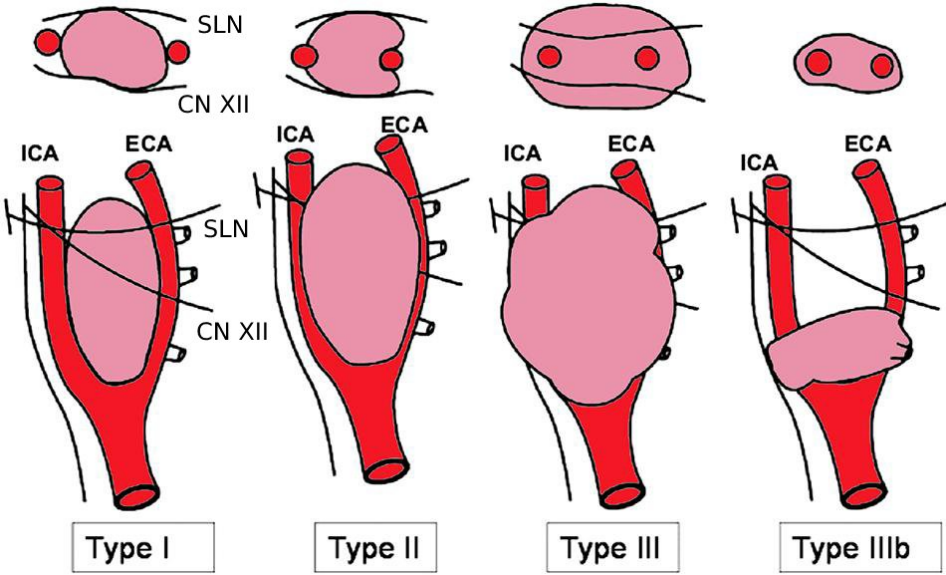
Shamblin classification (modified by Luna-Ortiz)

The hybrid treatment for carotid body tumor is highly indicated in the case of a vascularized tumor that involves the adjacent nervous structures: the type of tumor is discriminating, not only the Shamblin classification (Fig. 1).

The procedure involves the selective catheterization of the external carotid artery by transfemoral puncture, with the search for the vessel that contributes most to the tumor supply.

A branch of the ascending pharyngeal artery which irrigates the tumor is often associated with the glomus artery at the beginning of the mandibular bifurcation angle. Therefore, embolization of the external carotid artery is indicated, which often leads to mandibular claudication as a complication due to the temporary ischemia of the masseter muscle or the tongue. Embolization is performed by gelfoam or dacron particles. Recently the use of endovascular coils or spirals has been introduced for the same purpose.

Surgery is preferentially performed within 24-48h from embolization: in the surgical field the mass is so clearer and of a hard-elastic consistency, due to phenomena of ischemia and reactive fibrosis.

Preoperative embolization was introduced recently, as an additional technique, thanks to the progression of interventional radiology [18].

Pre-operative endovascular embolization is indicated in particular for CBT larger than 4 cm., or in case of tumor located cranially at the level or above the vertebral region C2.

The procedure is performed in conscious sedation, and requires expert anesthesiology assistance [19,20].

Femoral access is used to introduce a 5F catheter into the common carotid artery. The external carotid artery (ECA) is cannulated and then the arterial blood supply of the carotid body tumor are precisely defined. A catheter is selectively advanced to determine the smaller arteries that bring nourishment to the glomus tumor. Particles of polyvinitinol alcohol, mixed at 50% (1: 1 ratio) with contrast agent are then injected through the micro catheter to perform microembolization.

Initially, very small particles are injected (150-250 micrometers), so gradually larger particles, even greater than 1000 micrometers, are used to completely stop both the flow in the arterial branches and the outflow of the contrast medium. In this way, all the branches that bring nourishment to the glomus tumor are embolized.

Finally, an angiography is performed to evaluate the degree of success of the treatment: the result is considered satisfactory when there is a reduction of the blood supply to CBT greater than 50%

## 4 Methods

The aim of the study was to perform a comparative analysis of the short and medium-term results and complications between the traditional surgical approach and the hybrid procedure in the treatment of carotid body tumor. The study has complied with the rules of Ethics, consent and permits provided for studies involving human participants.

In a randomized study, 33 patients came to observation of the authors at Federico II University of Naples and at Mater Domini University of Catanzaro: they were enrolled between January 2004 and December 2016; 10 patients (pts.) were assigned to hybrid procedure, that is preoperative embolization and surgical resection of CBT (PE group), and 23 pts. to isolated traditional surgery (N-PE group).

**Table 1:** Demographic and glomic tumor variables in PE group (Preoperative Embolisation) and N-PE group (Non-Preoperative Embolisation: isolated traditional surgery)

VARIABLES	PE	N-PE	TOTAL	P-VALUE
Patients	10	23	33	…
Resections	11	24	35	…
Medium age	43-50	42-53	47	0,58
Males	3	10	13	0.12
Females	7	13	20	0.12
Bilateral tumors	5	5	10	0,1
Vagal Tumors	1	2	3	>0.99
SDHx mutation	2	1	8	0,1
Diameter cm	4,7	4,1	4,3	0,15
Volum cm3	26,2	25,2	25,5	0.81
Shamblin II	7	16	23	0,50
Shamblin III	4	7	11	0,50
Metastasis	0	1	1	>0,99
Familiarity	3	3	6	0,3
Tumor on the right	3	8	11	0,65
Tumore on the left	4	15	19	0,13

The study consists of 33 cases of carotid body tumors (14 males and 19 females), aged between 42 and 71 years (average age 59 years). Of these 22 pts. were smokers (13 males and 9 females); 5 pts. (3 males and 2 females) presented diabetes on oral antidiabetic therapy and 2 (1 male and 1 female) were insulin-dependent diabetics; 27 pts. (11 males and 16 females) were on therapy with antihypertensive drugs.

In 16 cases (7 males and 9 females) the enrolled patients reported repeated episodes of vertigo associated with hypertension (up to referred values of 190 mmHg of systolic pressure) and cold sweat: this symptoms generally persisted for about 20 minutes, to then regress spontaneously. Another 12 pts. (4 males and 8 females) were asymptomatic: in this group the diagnosis was based on the presence of a painless swelling, in a deep anterolateral cervical area. In 4 cases (2 males and 2 females) the initial diagnosis was incidental during an ultrasound examination of the neck, while in 1 case the diagnosis was derived from a PET-CT scan for oncological follow-up.

**Table 2:** Comparative analysis of surgical procedures in PE group (Preoperative Embolisation) and N-PE group (Non-Preoperative Embolisation: isolated traditional surgery)

VARIABLES	EMB	NEMB	TOTAL	P-VALUE
Patients	10	23	33	...
Open resection	1	2	3	>0.99
Nasotracheal intubation	2	4	6	>0,99
Parotid mobilization	2	5	7	>0,99
Mandibular subluxation	2	1	3	0,13
Interposition of saphenous vein	1	2	3	0,43
Protesic Patch	0	1	1	0,3
Term-terminal anastomosis	0	1	1	>0,99
ICA clamping	2	9	11	0,4
ECA ligation	1	5	6	0,26
ICA ligation	1	4	5	>0,99
EEG monitoring	10	22	32	0,9
Laryngeal nerve monitoring	1	o	1	0,93
Brain flow monitoring	1	4	5	0,16
Doppler monitoring	3	6	8	0,8

In all the cases of this study, the diagnosis was concluded with a CT-scan with contrast medium, which allowed the classification described by Shamblin: 20 pts. of our series (6 males and 14 females) were classified as Shamblin II and 13 pts. (4 males and 9 females) were classified as Shiamblin III type.

Patients were randomized by selecting enrolled patients for Group PE (Preoperative Embolization) and Group N-PE (Non-Preoperative Embolization), by a computerized randomization program with audit trial. Randomization was managed by the coordinator for randomization at Department of Medical and Surgical Science, University Magna Graecia of Catanzaro. Randomization was also indicated in the patient record.

The first group includes patients undergoing preoperative embolization, consisting of 3 males and 7 females. The size of the tumor oscillated between a diameter of 1.7 and 8 cm, with a maximum volume of 26.2 cubic cm. Among these, 6 CTB belonged to the Shamblin II group, the remaining ones to Shamblin III. The tumor occurred bilaterally in 44% of patients (P value = 0.1) and was of familial type in 34% (P value = 0.1); in the other cases, the tumor monolateral with prevalence on the left. About etiology, SDHx mutation was found in 25% of cases. No patient in the group had metastases at the time of diagnosis.

**Table 3:** Comparative analysis of intra- and post-operative complications in PE group (Preoperative Embolisation) and N-PE group (Non-Preoperative Embolisation: isolated traditional surgery)

VARIABLES	EMB	NEMB	TOTAL	P-VALUE
Patients	10	23	33	...
Cranial nerves resection	1	2	3	0,72
EEG Changes	0	2	2	>0.99
Bradycardia-ipotension	0	1	1	>0,99
Operative time (minutes)	250	265	259	0,49
Blood loss(mL)	263	599	486	0,02
Respiratory failure	0	1	1	>0,99
Hematoma	0	1	1	>0,99
Infections	0	1	1	>0,99
ICA occlusion	0	1	1	>0,99
Temporary damage of the cranial nerves	6	9	15	0,21
Permanent cranial nerves damage of the	1	2	3	0,46
Post-(days) operative hospitalization	4,1	4,2	4,1	0,91

Preoperative embolization procedure was performed with trans-femoral access, with a 5F catheter placed in the common carotid artery. A micro catheter was then introduced into the ECA, and particles of alcohol and polynitinol (up to 250 micrometers in size) were introduced to achieve embolization.

The second group of patients (N-PE) were treated without the preoperative embolization procedure and included 11 males and 12 females. The carotid body tumor in these cases had a diameter between 2.5 and 7 cm., with a maximum volume of 25.2 cubic cm. 60% (14 cases) of these cases were classified as Shamblin II, and the remaining 40% (9 cases) as Shamblin III.

Bilateral carotid body tumors were found in 22% of cases and familiarity in 14% of cases; monolateral tumors, on the other hand, as reported the first group, were found with a higher prevalence on the left side. Only a small part of the patients (2 cases) had SDHx mutation. Only one case of metastasis was identified at diagnosis, with PET-TAC total body.

**Table 4:** Results of the logistic regression model for evaluation of temporary damage of cranial nerves. 2004-2016 serie.

VARIABLES	ODDS RATIO	95% CONFIDENCE INTERVAL	P-VALUE
Age, tumor growth in 10 years	0,97	(0,79-1,19)	0,74
Male sex	0,81	(0,39-1,66)	0,56
Bilateral tumor	1,05	(0,5.2,22)	0,9
No familiarità	0,75	(0,34-1,69)	0,49
Familiarity	0,87	(0,38-2,02)	0,74
SDHx mutations	0,6	(0,21-1,76)	0,35
Pre-operative embolization	3,14	(1,42-6,96)	0,04
ASA 1	1	1	...
ASA 2	0,41	(0,13-1,23)	0,11
ASA 3	0,71	(0,25-2,03)	0,52
Simple excision	2,19	(0,60-7,98)	0,23
Complex excision	2,25	(0,74-6,84)	0,15
ICA clamping	1,51	(0,70-3,26)	0,3
Operative time in minutes	2,69	(1,41-5,13)	0,03
Tumor sizes in cm3	1,51	(1,16-1,98)	0,03
Shamblin I	1	1	...
Shamblin II	6,7	(1,88-23,95)	0,03
Shamblin III	16,74	(4,28-65,48)	<0,01
Loss of blood mL	1,44	(1,12-1,85)	0,05

The surgical risk of cranial nerve injury and blood loss were communicated with greater scrupulousness to all patients before they underwent f surgery, during the collection of the Informed Consent to surgical intervention.

Surgical resection was performed in all cases by incising the anterior edge of the sternocleidomastoid muscle, isolating the vagus, hypoglossal and superior laryngeal nerves and proceeding to remove the tumefaction in the cranio-caudal direction.

**Ethics approval and consent to participate**: All patients underscribed an informed consent to be included in the study. Ethics committee approval was not necessary as the technique was already considered a standard procedure in our Hospital.

**Consent for publication**: The patients gave written informed consent to publish.

## 5 Statistic analysis

The data obtained were evaluated to find statistical significance using the p-value and hypothesis test,. The patients were divided into two groups, each characterized by several variables, independent of each other.

The comparative analysis was carried out using the Fisher, Chi square, Wilcoxon rank-sum and Kruskal Willis tests. The association of the (permanent and reversible) damage assessment of cranial nerves was evaluated with a logistic regression and summary model using the ODDS RATIO and 95% confidence intervals. Because of inhomogeneity of some data, they have been transformed using log [2] for the regression model. All the data were then compared, as already mentioned, using the P-value test: the data was considered statistically significant, supporting our hypothesis if the P-value was lower than 0.5.

## 6 Results

**Figure 2:**
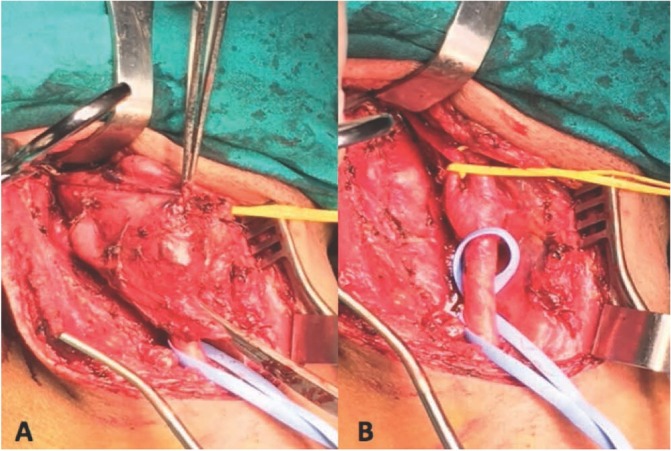
Intra-operative pictures during surgical excision of a CBT of PE-group (treated with Pre-operative Embolization ) : A) pre-excision; B) post-excision.

In surgical procedure to CBT in patients of theN-PE group, nose-tracheal intubation was used in 5 cases (22%); surgical procedure involving the mobilization of the parotid gland and the mobilization of the mandible occurred in 3 cases (10%). In the PE group, such procedures were used only in 15% of cases. (22% v/s. 15%, P value = 0.3). In addition, patients of the N-PE group had to use a saphenous vein interposition in 2 cases and a prosthetic patch in one case. In the hybrid procedure (PE group), both less use of external carotid clamping (15% v/s 37%; P value = 0.3), and its ligation (10% of cases) were observed. On the contrary, both procedures of clamping and ligation were more frequently necessary in N-PE group, where occurred also one case of temporary clamping of common carotid. During surgery all patients were all equally monitored by electroencephalography. In the hybrid procedure attention was paid especially to the monitoring of the recurrent laryngeal nerve (10%), considered superfluous for the N-EP group, where greater attention was paid for cerebral flow monitoring (Fig. 2 intraoperative surgical filed in PE).

Intraoperative complications were far lower in patients of PE group: injury of cranial nerves were reported in 7% of cases compared to 12% of the N-PE group (P-value = 0.72); while reversible nerve lesions (P value = 0.21) and the permanent ones (P value = 0.46), were found similar in both groups: this information contrasts with the lower incidence of nerve resection that occurs in the first group. Exactly for this reason, the aforementioned complication was also evaluated in relation to the classification of treated tumors (Shamblin II, prevalent in the N-PE group and Shamblin III prevalent in the EP group), using ODDS RATIO and 95% confidence interval.

Regardless of the surgical procedure performed the reversible damage of the cranial nerves occurred with lower incidence in Shamblin III patients (OR = 16.74; confidence interval 4.28-65.48, P-value <0.01) in cases treated with a PE procedure. Shamblin II patients, predominantly treated with N-PE, had a fairly low reversible nerve damage incidence (OR = 6.70; 1.88-23.95 confidence interval, P value = 0.03). The difference between the two P values is not statistically significant, therefore, regardless of the procedure, the reversible damage to the cranial nerves is similar. This is very different for nerve resection that is affected by the size as well as the proximity to nerve structures, particularly in the Shamblin III tumors, so nerve damage was greater for the N-EP group. Bradycardia and hypotension remain, on the other hand, comparable complications in both procedures (P-value 0.99). Also the electroencephalogram alterations during the surgical procedure seem to confirm greater safety of hybrid procedure in the treatment of CBT (7% v/s. 13%; P value> 0.99). Great importance for the confirmation of the validity of the hybrid treatment in the treatment of CBTs lies in the evaluation of blood loss during surgery. The comparison between the two procedures shows a P-value of 0.02 which indicates a significant difference between the first group and the second group: in PE group the loss of blood is less than 50% of that of the N-PE group (263 mL v/s 599 mL), which further confirms the importance of using a hybrid procedure for widely vascularized tumors, according with Shamblin classification.

Also with regard to long-term complications, such as respiratory failure, infections, ICA obstruction, and stroke, we did not report significant differences between the EP group and the N-EP group (P value> 0.99). The formation of a hematoma instead occurred only in one of the patients treated in the N-PE group (P-value = 0.54). At the same time, there was no difference with regard to postoperative hospitalization times, estimated for both groups at about 5 +/- 2 days (P-value = 0.91), but follow-up time for patients of the group EP were shorter (26 months versus 48 months of traditional surgery P value = 0.3).

Ultimately, the reported data shows that in the EP group, a less invasive surgical procedure is required compared to N-EP (97% v/s 82%; P value = 0.3). There is also a lower incidence of ECA clamping (15% v/s 37%; P value = 0.4) in the EP group. Finally, the loss of blood, as already highlighted above, is lower in the EP group (P value = 0.02): the P value indicates in this case a significant difference between the two group of patients, giving value to hybrid treatment of CBTs.

On the other hand, operating times (250 v/s 265 minutes, P value = 0.49), reversible damage of the cranial nerves (52% v/s 38%, P value = 0.21), operative mortality and early postoperative (absent in both procedures, P value> 0.99), the incidence of stroke (0% v/s 1%, P value> 0.99), the post-operative hospital stay (4.1 v/s. 4.2 days, P value = 0.91) are not different between the two procedures described.

The analysis of the results shows that pre-operative embolization of CTB reduces intraoperative blood loss and resection of the cranial nerves; the procedure also allows a less invasive operation to be carried out, thereby reducing the follow-up period.

## 7 Discussion

The data results demonstrated the superiority, although not significant due to the low number of samples, of pre-operative embolization in reducing hemorrhagic complications during surgical CBT resections, in terms of blood loss requiring transfusions, and moreover in reducing ischemic complications, in terms of need for clamping or ligation of branches of the carotid artery, but above all the benefit on nervous complications in terms of reduction of incidence, severity and duration of nervous dysfunctions induced by surgery. Pre-operative embolization of CBT, as a single or repeated procedure, with a short interval of time (24-48 hours) before surgical resection of tumors of greater size (> 4 cm.), did not show negative effects, such as inflammation and tissue necrosis, or specific complications (infection or bleeding of the puncture site, cervical or cerebral edema, spasms, embolization or ischemic brain tissue), and is able to modify the immediate and long–term results of that surgery.

Finally, the results of this study validate the hypothesis, also supported in the experiences of other authors [21, 22, 23] of the validity of the pre-operative embolization of CBT.

However the present study has some limitations: it has a relatively small number of patients, and excludes the lack of a long-term follow-up for all patients.

## 8 Conclusions

Preoperative embolization in the case of resection of the carotid body tumors is favorable and advisable in all cases of surgical planning for the treatment of carotid body tumors, particularly in cases of larger and involving large-scale vascular and nervous structures.

This procedure, according to the results presented in this article, can therefore be validated for the purposes of scientific society guidelines and to be considered as a contribution to the guidelines in that field.

## List of abbreviations

ECA =: external carotid artery
CTB =: carotid body tumor
PE =: preoperative embolization
N-PE =: non-preoperative embolization
